# Cryogenic 3D printing of dual-delivery scaffolds for improved bone regeneration with enhanced vascularization

**DOI:** 10.1016/j.bioactmat.2020.07.007

**Published:** 2020-08-12

**Authors:** Chong Wang, Jiahui Lai, Kai Li, Shaokui Zhu, Bingheng Lu, Jia Liu, Yujin Tang, Yen Wei

**Affiliations:** a School of Mechanical Engineering, Dongguan University of Technology, Songshan Lake, Dongguan, Guangdong, PR China; b Department of Mechanical Engineering, The University of Hong Kong, Hong Kong SAR, PR China; c Department of Orthopedics, The Third Affiliated Hospital of Southern Medical University, Guangzhou, Guangdong, PR China; d Department of Orthopaedics, Affiliated Hospital of Youjiang Medical University for Nationalities, Baise, Guangxi, PR China; e Department of Chemistry, Tsinghua University, Beijing, PR China

**Keywords:** Cryogenic 3D printing, Dual-delivery, Osteogenesis, Angiogenesis, Bone regeneration

## Abstract

Three-dimensional (3D) printing has been increasingly employed to produce advanced bone tissue engineering scaffolds with biomimetic structures and matched mechanical strengths, in order to induce improved bone regeneration in defects with a critical size. Given that the successful bone regeneration requires both excellent osteogenesis and vascularization, endowing scaffolds with both strong bone forming ability and favorable angiogenic potential would be highly desirable to induce improved bone regeneration with required vascularization. In this investigation, customized bone tissue engineering scaffolds with balanced osteoconductivity/osteoinductivity were produced via cryogenic 3D printing of β-tricalcium phosphate and osteogenic peptide (OP) containing water/poly(lactic-*co*-glycolic acid)/dichloromethane emulsion inks. The fabricated scaffolds had a hierarchically porous structure and were mechanically comparable to human cancellous bone. Angiogenic peptide (AP) containing collagen I hydrogel was then coated on scaffold surface to further provide scaffolds with angiogenic capability. A sequential release with a quick AP release and a slow but sustained OP release was obtained for the scaffolds. Both rat endothelial cells (ECs) and rat bone marrow derived mesenchymal stem cells (MSCs) showed high viability on scaffolds. Improved *in vitro* migration and angiogenesis of ECs were obtained for scaffolds delivered with AP while enhanced osteogenic differentiation was observed in scaffolds containing OP. The *in vivo* results showed that, toward scaffolds containing both AP and OP, the quick release of AP induced obvious angiogenesis *in vivo*, while the sustained OP release significantly improved the new bone formation. This study provides a facile method to produce dual-delivery scaffolds to achieve multiple functions.

## Highlights

• Dual-delivery scaffolds are structurally and mechanically similar to cancellous bone.
• Dual-delivery scaffolds can sequentially release angiogenic and osteogenic peptides.
• Dual-delivery scaffolds improve bone regeneration and vascularization *in vivo*.

## Graphical abstract

**Figure:**
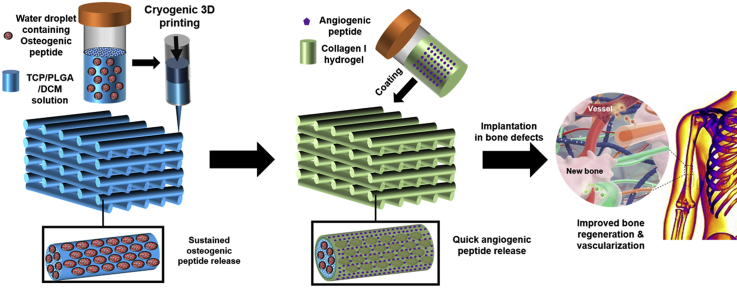

## 1 Introduction

Three-dimensional (3D) printing has become a versatile platform technique to greatly improve our capability to fabricate complex tissue engineering scaffolds with tailored structural, mechanical, physical/chemical and biological features by accurately positioning biomaterials, biomolecules and even live cells in allocated area in a layer-by-layer manner [[1], [2], [3]]. By using a variety types of 3D printing technologies such as fused deposition modelling [4], selective laser sintering [5], robotic dispensing [6], etc., bone tissue engineering scaffolds with tunable mechanical strengths, controllable degradation rate and adjustable biological performances can be produced from polymer melts, bioceramic powders, polymer/bioceramic composite wires/pellets and ceramic containing hydrogels [[7], [8], [9], [10]]. Among different biomaterials, calcium phosphate bioceramics are the most used ingredient in making osteoconductive bone tissue engineering scaffolds [11,12], but scaffolds made of bioceramics alone or even polymer/bioceramic composites lack of sufficient bone forming ability. Given that growth factors are capable of directing stem cell differentiation and accelerate tissue/organ regeneration, incorporating growth factors in scaffolds to achieve a sustained delivery is highly favorable [13]. Among various growth factors, bone morphogenetic protein-2 (BMP-2) is the most potent biomolecule to induce osteogenic differentiation of mesenchymal stem cells (MSCs) *in vitro* and bone formation *in vivo* [14,15]. In recent years, cryogenic 3D printing has attracted increasing attention and is being applied to develop bone tissue engineering scaffolds with suitable mechanical properties, biomimetic hierarchical porous structures and *in situ* delivery of biomolecules, for enhanced cell responses and bone formation *in vitro* and *in vivo* [[16], [17], [18]]. Nevertheless, bone tissue engineering scaffold for clinical use should not only possess excellent osteogenesis for new bone formation but also has interconnected vasculatures for nutrients transfer, oxygen exchange, wastes clearance, cells and signal molecules regulation [7,19]. Despite numerous types of 3D printed bone tissue engineering scaffolds have been constructed and studied *in vitro* and *in vivo*, it is still challenging to produce a bone tissue engineering scaffold to enhance the vascularization during new bone formation [19]. It is known that vascular endothelial growth factor (VEGF) is an effective growth factor to improve the scaffold vascularization via angiogenesis [20]. Therefore, the combined delivery of multiple types of growth factors in scaffolds becomes one useful approach to improve osteogenesis with required angiogenesis. Besides, the release kinetics of different biomolecules within the 3D printed scaffolds should be carefully controlled to effectively promote the formation of vasculature and new bone within the scaffolds. Park *et al.* fabricated a dual-growth factor delivery scaffolds containing BMP-2 and VEGF via 3D printing [21]. This scaffold could control the spatial and temporal release of BMP-2 and VEGF to promote the repair of large bone defects. However, their work failed in fabrication of porous hierarchical structures mimicking natural bone, which is also very vital for improved bone repair. Therefore, it is highly important to fabricate scaffolds which not only have tailored shape and biomimetic architectures to mimic natural bone but also have the capability to deliver multiple types of biomolecules to stimulate angiogenesis and osteogenesis during bone regeneration process. Although BMP-2 and VEGF are commonly used growth factors for osteogenesis and angiogenesis, they are unstable macromolecules and expensive. Osteogenic peptide (OP) and angiogenic peptide (AP) are synthesized peptides which are functionally equivalent to BMP-2 and VEGF, respectively. OP and BMP-2 bind the same receptor to up-regulate the signaling pathway of osteogenesis while AP and VEGF also bind the same receptor (different from the receptor binding OP or BMP-2) to launch the signaling pathway of angiogenesis. As OP and AP can be synthesized in a large scale, they have a much lower cost and hence a larger amount of peptides can be used to better improve the osteogenesis and angiogenesis. The use of AP and OP have also been reported by some studies [22,23].

In this study, customized bone tissue engineering scaffolds with excellent mechanical properties, hierarchically porous structure and balanced osteoconductivity and osteoinductivity were produced via cryogenic 3D printing of β-tricalcium phosphate and OP containing water/PLGA/DCM emulsion inks. The coating of AP containing collagen hydrogel on the scaffold surface further endowed scaffolds with the potent of vascularization. The dual delivery of OP and AP in the strut matrix and surface coating, respectively, enabled a sequential release of AP and OP. The biological performance of the scaffolds was studied. The capability of angiogenesis and osteogenesis of the scaffolds was investigated *in vitro* and *in vivo*, demonstrating that the customized scaffolds with dual delivery of osteogenic/angiogenic peptides could greatly improve the bone regeneration and enhance the vascularization.

## 2 Methods

### 2.1 Materials

Poly(lactic-*co*-glycolic acid) (PLGA) with a LA:GA ratio of 50:50 and a molecular weight of 100,000 was supplied by Jinan Daigang Biomaterials Ltd, China. β-tricalcium phosphate (β-TCP) with a diameter of 200 nm was supplied by Shanghai Aladdin Reagent Co Ltd, China. Osteogenic peptide (OP) with a sequence of KIPKA SSVPT ELSAI STLYL SGGC and a purity of 98.12% and angiogenic peptide (AP) with a sequence of KLTWQ ELYQL KYKGI and a purity of 98.12% were provided by Shanghai Ziyu Biotechnology Ltd, China. Rat tail derived collagen I solution with a concentration of 8.9 mg/mL was a Corning product (USA). Deionized (DI) water was prepared by an ultra-pure water system (Arum 611, Sartorius).

### 2.2 Fabrication of TCP/PLGA scaffolds spatially loaded with AP and OP

To achieve improved bone tissue engineering with required vascularization, dual-peptide delivery scaffolds were fabricated. Firstly, 3 g of PLGA was dissolved in 10 mL of DCM, followed by the addition of 3 g of TCP particles. With the assistance of 30 min of ultra-sonication in an ice water bath, uniform TCP/PLGA/DCM suspension was obtained. Subsequently, 2.5 mL of DI water containing 10 mg of OP was added into the TCP/PLGA/DCM suspension, followed by a manual stirring for 20 min to obtain water-in-oil composite emulsions with a color in white. The as-prepared emulsions were used as inks and loaded in a 20 mL syringe which was connected with a V-shape nozzle (inner diameter of 0.4 mm). A pre-designed STL file was imported in a cryogenic 3D printer and scaffolds with 3D grid pattern were printed. After the cryogenic 3D printing, as-fabricated scaffolds were freeze-dried to remove water/DCM, thus stabilized bone tissue engineering scaffolds can be obtained. To further endow scaffolds with the capability for angiogenesis, 100 mg of scaffold samples were then coated with 0.1 mL of collagen I working solution (5 mg/mL) containing 0.5 mg of AP, followed by a gelling treatment for 30 min at 37 °C. The coated scaffolds were dried at room temperature to obtain bone tissue engineering scaffolds with dual-peptide delivery capability.

### 2.3 Scaffold characterization

Digital camera and scanning electron microscopy were used to observe the macro- and microstructures of different scaffolds. Compression testing was used to study the mechanical properties of dual-peptide delivery scaffolds and other controls. Typically, scaffold samples with a dimension of 10 mm × 10 mm × 10 mm were immersed in PBS solution and then subjected to a compression testing using a universal testing machine at 37 °C. The strain rate was set as 2 mm/min and the compression was stopped when 50% compression strain was achieved. 5 samples for each type of scaffold were tested. The compression strength and elastic modulus were calculated.

### 2.4 *In vitro* release and degradation

To study the *in vitro* release behaviour of two types of peptides (i.e., AP and OP), AP-RhB and OP-FITC which were grafted with Rhodamine B and FITC, respectively, were used to replace AP and OP to fabricate scaffolds with dual-peptide delivery. 50 mg of AP-RhB and OP-FITC loaded scaffold sample and other controls were separately added into 15 mL centrifuge tubes which were further added with 3 mL of PBS solution (pH 7.4, added) supplemented with 0.02% (w/v) sodium azide. The tubes were then put in a shaking water bath and the temperature was maintained at 37 °C. During a 42-day test period, at pre-determined time intervals, the release test liquid was taken out and the concentration of AP-RhB and OP-FITC in the test liquid was measured using a fluorescence microplate reader. Afterwards, fresh PBS solution with the same volume was added in the tubes for continuous incubation. The *in vitro* degradation behaviour of scaffolds was studied by monitoring the weight remaining in an 8-week test period. Typically, 100 mg of scaffold sample was put into a 15 mL centrifuge tube which was added with 3 mL PBS solution. The test liquid was changed every week. After 2-, 4-, 6-, and 8-week of incubation, the scaffold samples were taken out and rinsed in DI water for several times to remove precipitated salts. The rinsed scaffolds were then freeze-dried for 48 h and the weight remaining was measured using a digital balance.

### 2.5 *In vitro* growth, migration and vascularization of rECs

A rat endothelial cell (rEC) containing vial (Life Technologies, NY, USA) was thawed and expanded in Medium 200 (Life Technologies, NY, USA) supplemented with 1% (v/v) Low Serum Growth Supplement, 100 U/ml P/S and 2 mM l-glutamine (Life Technologies, NY, USA) in a 37 °C incubator pumped with 5% CO_2_/95% air. The EC migration was studied using a wound healing method. 1 ml of cell suspension with a density of 5 × 10^4^ cells/mL was dripped in wells of a 48-well plate and then filled with 400 ml of culture medium. Once rECs achieved 80% confluence, a 200 μL micropipette tip was used to scratch on the cells to form a 400 μm wide wound and dissociated cells were removed. Afterwards, 400 μL of culture medium was supplemented in each well and a pre-weighed scaffold sample (5 mg) was added into the well. The scaffold samples floating in the culture medium released agents and there was no contact between scaffold samples and cells. After 12 h of incubation, the distance of rEC migration was measured under a microscope. To study the *in vitro* vascularization, 50 μL of Matrigel (4 °C, Corning, USA) was added into a well of a 96-well plate and incubated for 30 min at 37 °C. Afterwards, 100 μL of rEC cell solution containing 3 × 10^4^ rECs was added into the well, followed by the addition of a scaffold sample with a weight of 5 mg. The scaffold samples were floated in the medium. After 12 h of culture at 37 °C, the scaffold samples were removed and the formed rEC tube networks were observed using a microscope.

### 2.6 *In vitro* growth and osteogenic differentiation of rBMSCs

A rat bone marrow derived mesenchymal stem cell (rBMSC) containing vial was thawed and expanded in Dulbecco's modified Eagle's medium/F-12, (DMEM/F-12, Gibco, USA) which was supplemented with 10% fetal bovine serum (Gibco, USA) and 100 U/ml penicillin-streptomycin and maintained in a humidified incubator at 37 °C with 5% CO_2_. The medium was changed every 2 days. Afterwards, 100 μL of rBMSCs with a density of 1 × 10^5^ were seeded on wet scaffolds, followed by the addition of 400 μL of medium after 4 h of culture. The viability of rBMSCs on scaffolds was examined on day 3 using a live and dead assay. To investigate the osteogenic differentiation, alkaline phosphatase (ALP) staining was used to stain rBMSCs to visualize the ALP^+^ area (ALP staining kit, Beyotime Biotechnology Ltd, China). ALP activity of rBMSCs on scaffolds was also investigated using an ALP activity assay (Beyotime Biotechnology Ltd, China). The extracts of scaffolds were also supplemented into the osteogenic media to culture cells for 21 days and the cell mineralization was visualized via Alizarin Red S (ARS) staining. After taking micrographs, 10% cetylpyridinium chloride was used to dissolve the nodules, and the absorbance was examined at 562 nm.

### 2.7 Construction of cranial defect model and scaffold implantation

All animal surgical procedures were conducted under protocols approved by the Committee on the Use of Live Animals in Teaching and Research, Dongguan University of Technology. 25 male Sprague-Dawley rats (10 weeks old, body weight: 300–350 g) were used for experiments. The rats were anesthetized by intraperitoneal injection of pentobarbital, then a sagittal incision of 1.5–2.0 cm was made on the scalp. One full-thickness rectangular defect (5 mm × 3 mm) was made on one side of craniums using an electric trephine drill. Then different scaffolds (n = 5 for each group) were cut into implants with a dimension of 5 mm × 3 mm and a thickness of 1 mm, and implanted into cranial defects afterwards. The soft tissues were re-positioned and sutured with 4-0 silk sutures to achieve primary closure. Sham surgery (i.e., defect implanted with no scaffolds) was also conducted and used as control group. Each rat received an intraperitoneal injection of antibiotics post-surgery. Micro-CT evaluation and corresponding histomorphometric analyses were conducted after two and three months, respectively. After 3 months post-surgery, 25 rats were sacrificed, and obtained calvarias were scanned using micro-CT (Skyscan 1176, Kontich, Belgium). The scanning was performed at a resolution of 18 μm and the images were acquired to reconstruct tomograms with 3D Creator software. The ratio of bone volume/tissue volume (BV/TV) and bone mineral density (BMD) were measured using CTAn image analysis software based on the micro-CT images.

### 2.8 Histological analysis

Cranias were retrieved, fixed, decalcified, dehydrated and defatted before embedding in paraffin, as previously described. Then, the samples were cut into 4 mm thick sections by a paraffin microtome (RM2125RTS, Leica, Wetzlar, Germany). The sections were stained following Masson's trichrome staining methods in accordance with the standard protocols. For all types of stained sections, bone histomorphometric analysis was performed under a semi-automated digitizing image analyzer system with a ZEISS Scope.A1 (ZEISS, Germany).

### 2.9 Statistical analysis

Quantitative data are expressed as mean ± standard deviation (s.d.). One-way ANOVA tests via Student's t-tests were conducted and the data were indicated with (*) for probability less than 0.05 (*p* < 0.05).

## 3 Results and discussion

### 3.1 Scaffold design

**Fig. 1:**
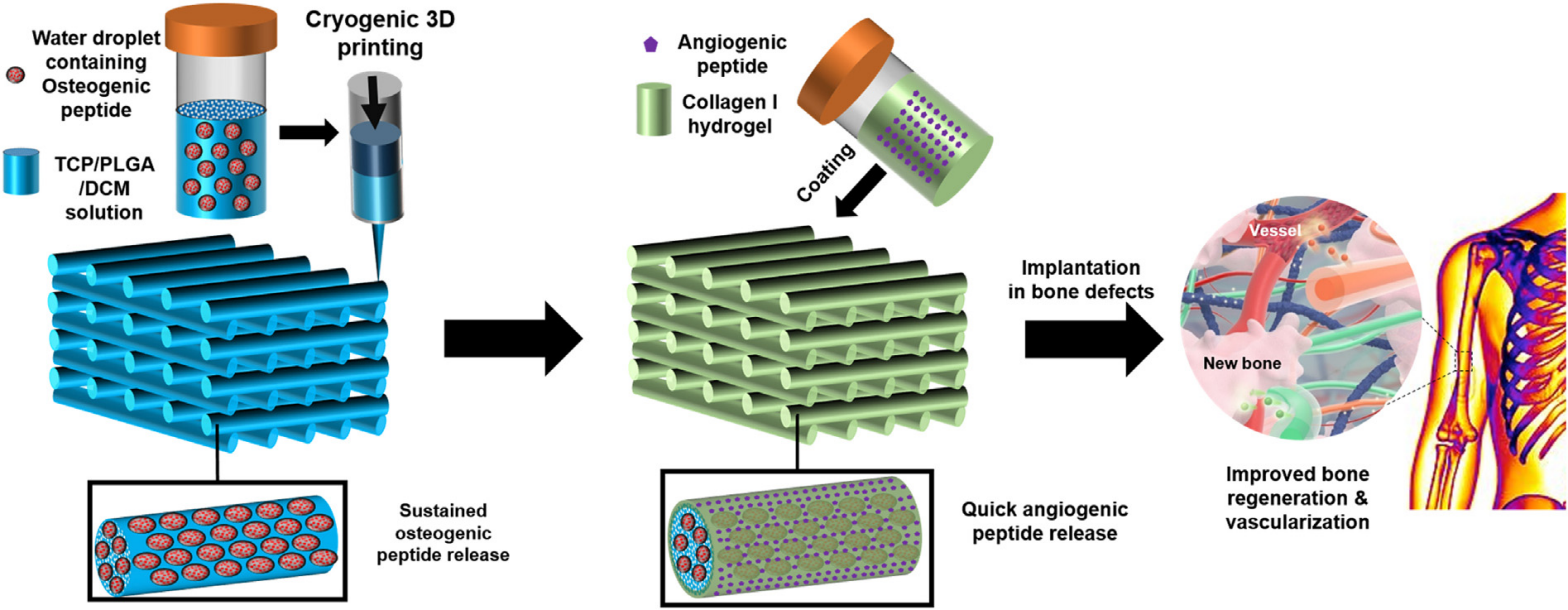
Schematic illustration of fabricating AP and OP delivered TCP/PLGA composite scaffolds via cryogenic 3D printing and subsequent hydrogel coating. The scaffold can be implanted in bone defects to induce improve bone regeneration with required vascularization.

Aiming at repairing/regenerating bone tissue at the defected site with a critical size, customized dual-delivery composite scaffolds comprising OP/TCP/PLGA struts and AP/collagen I coating were fabricated through cryogenic 3D printing, followed by the coating of AP/collagen I hydrogel precursor and subsequent gelling treatment (Fig. 1). The AP/collagen I hydrogel layer provides scaffolds with improved angiogenesis capability via a quick AP release from the collagen I hydrogel layer, whereas hierarchically porous OP/TCP/PLGA struts provide scaffolds with improved capability for *in vitro*/*in vivo* osteogenesis, through the sustained OP release from the bony microporous environment.

### 3.2 Scaffold characterization

**Fig. 2:**
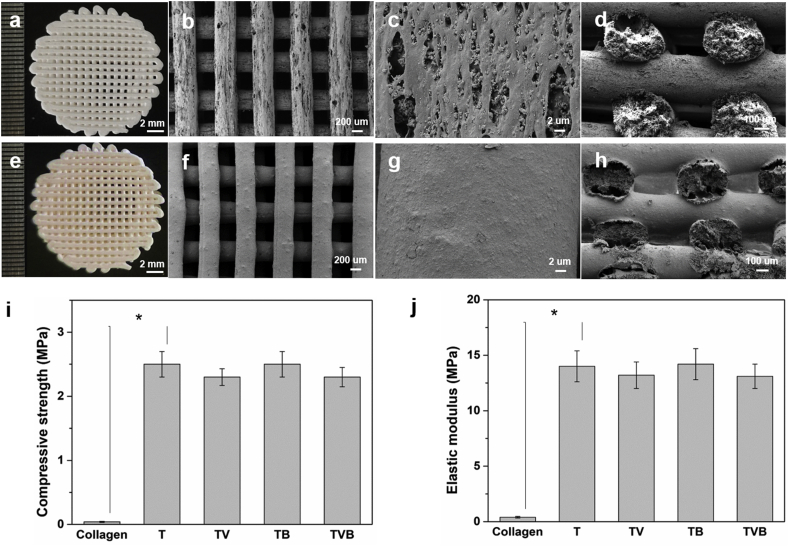
Morphology and mechanical properties of OP/TCP/PLGA and AP/collagen/OP/TCP/PLGA scaffolds. (a) and (e): digital images; (b-d, f-h): SEM micrographs of different scaffolds at different magnification; (i) compressive strengths of scaffolds and controls; (j) elastic modulus of scaffolds and controls. T, TV, TB, TVB refer to TCP/PLGA, AP/collagen/TCP/PLGA, OP/TCP/PLGA, AP/collagen/OP/TCP/PLGA, respectively.

Fig. 2 shows the morphology of OP/TCP/PLGA scaffolds with and without the coating of AP/collagen I hydrogel. As shown in Fig. 2a and e, both OP/TCP/PLGA scaffold and AP/collagen/OP/TCP/PLGA scaffold had a regular grid-like pattern. However, OP/TCP/PLGA scaffolds had a rough strut surface on which a number of micropores and numerous TCP particles (200 nm) can be observed (Fig. 2b and c) [24]. In comparison, after the coating of AP/collagen I hydrogel, most micropores were filled with hydrogels, and thus a relatively smooth strut surface can be observed (Fig. 2f and g). The cross-sections of two types of scaffolds are shown in Fig. 2d and h, respectively. No difference was observed, suggesting that even after the coating, the inner structure of the struts was not altered, showing a microporous morphology. The mechanical properties of different scaffolds and controls were also studied. As shown in Fig. 2i and j, collagen I hydrogel had a relatively low compressive strength (0.04 MPa) and elastic modulus (0.4 MPa). In comparison, T and TB scaffolds had a compressive strength and an elastic modulus of 2.5 MPa and 14.1 MPa, respectively, which are comparable to human cancellous bone [25]. It is also found that the further coating of collagen I hydrogel onto the bone tissue engineering scaffolds (i.e., TV and TVB scaffolds), only a slight reduction in the compressive strength and elastic modulus was observed, suggesting that cryogenic 3D printed bone tissue engineering scaffolds coated with a thin layer of collagen I hydrogel are mechanically suitable for repairing/regenerating defected bone tissue.

### 3.3 *In vitro* release and degradation behaviour

**Fig. 3:**
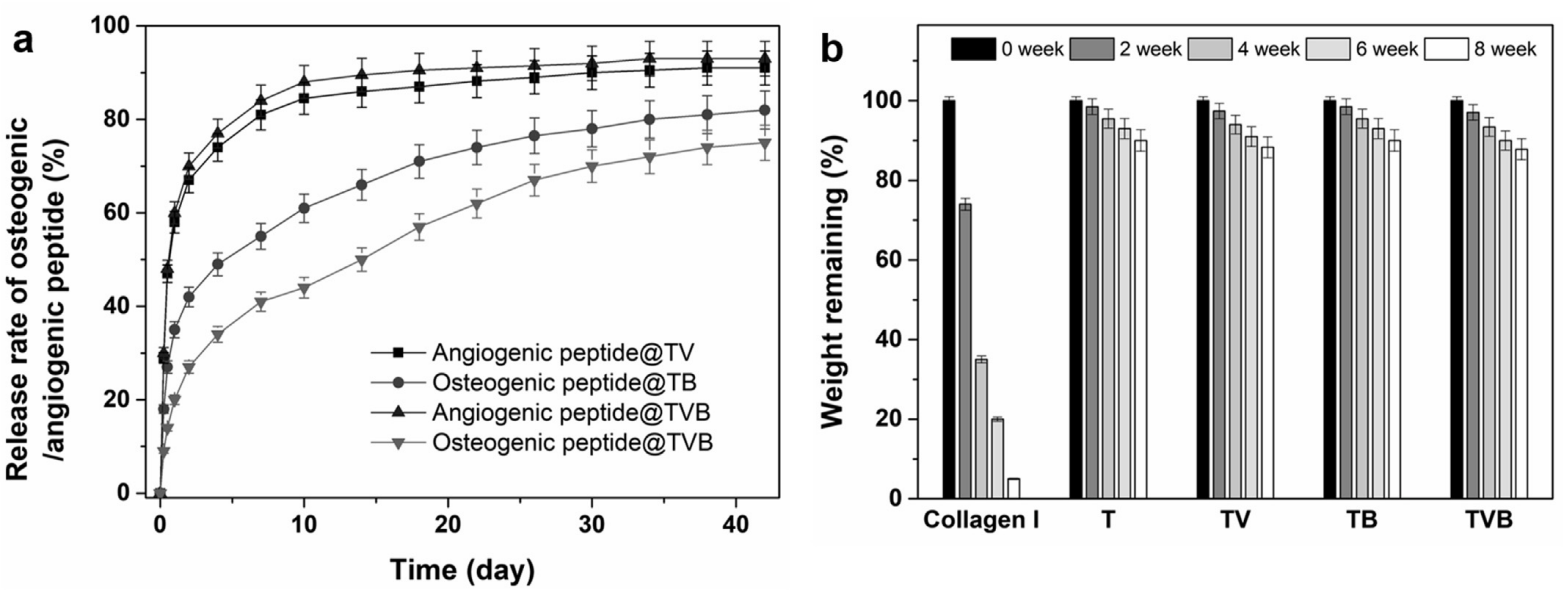
*In vitro* release behaviour and degradation behaviour of dual-peptide delivery scaffolds. (a) release behaviour of angiogenic peptide and osteogenic peptide in a 42-day test period; (b) weight remaining of dual-peptide delivery scaffolds and controls in an 8-week test period.

The *in vitro* release behaviour of two types of peptide from different scaffolds was also studied. As shown in Fig.3a, a quick AP release (up to 58% level) was observed in initial 24 h for TV and TVB groups, and the release profile achieved a plateau after 10 days of incubation. This could be attributed to the quick diffusion of AP from the hydrogel layer to the test liquid. In comparison, OP released from TB scaffolds exhibited a more sustained release profile by showing 35% release level in initial 24 h and a total release level of 79% in 42 days. It is worth noting that the OP released from TVB scaffolds was significantly lower than that released from TB scaffolds within 10 days. This could be attributed to the delayed diffusion of OP from the micropores of OP/TCP/PLGA struts to the test liquid, due to the barrier effect of collagen I hydrogel coating on the strut surface. However, when a part of collagen I hydrogel degraded, the diffusion of OP from the TVB scaffolds was accelerated to some extent and a OP release level up to 74% can be achieved in 42 days, which was very close to the total release level of OP from TB scaffolds at day 42.

The *in vitro* degradation behaviour of different scaffolds and collagen I hydrogel was studied by monitoring their weight remaining in an 8-week test period (Fig. 3b). Towards collagen I hydrogel, quick degradation was observed during the whole test period due to the rapid hydrolysis of collagen matrix in an aqueous environment, showing 25% and 95% weight loss after 2 and 8 weeks of incubation, respectively. In comparison, T and TB scaffolds showed a much less weight loss due to the much slower hydrolysis rate of PLGA in the test liquids. For TV and TVB scaffolds which contain both TCP/PLGA matrix and collagen I hydrogel coating, a slightly higher weight loss was observed, compared to that of T and TB scaffolds (*p* > 0.05), indicating that the dual-delivery scaffolds were stable enough to undertake the role for supporting long-term bone regeneration.

### 3.4 *In vitro* biological performance of scaffolds

**Fig. 4:**
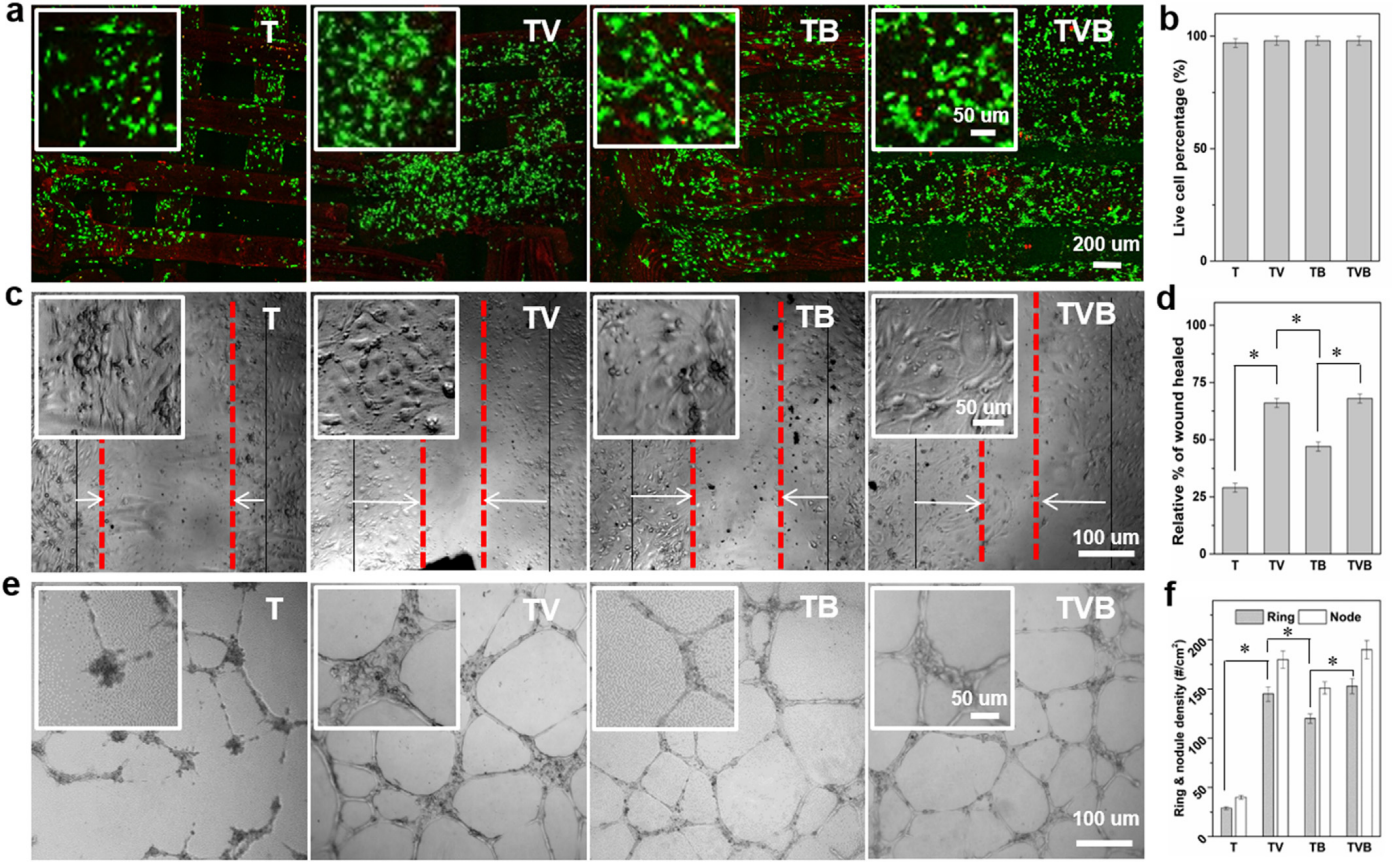
Angiogenic capability of scaffolds. (a–b) live and dead images of ECs on scaffolds and viability; (c–d) effect of extracts of different scaffolds on the EC migration after 12 h of culture; (e–f) effect of extracts of scaffolds on EC tube formation on Matrigel.

We then investigated the biological performance of the dual-delivery scaffolds. To study their capabilities of *in vitro* angiogenesis, rat endothelial cells (ECs) were used as model cells. As shown in Fig. 4a, nearly all ECs seeded on all scaffolds were alive after 3 days of culture, showing a very high viability (98%) (Fig. 4b). The effect of different scaffolds on the migration of ECs was studied via wound healing experiments. After 12 h of incubation, compared to T scaffolds, the extracts of TB scaffolds could significantly improve the EC migration. Moreover, extracts of TV and TVB scaffolds could further enhance the EC migration (Fig. 4c and d), indicating that the quick AP release is the key factor in the acceleration of EC migration. The effect of scaffolds on EC tube formation in Matrigel was also investigated. As shown in Fig. 4e and f, extracts of T scaffolds induced very limited tube formation, whereas extracts of TV, TB and TVB induced significantly more tube formation, not only in terms of the number of rings but also in terms of the number of nodes.

**Fig. 5:**
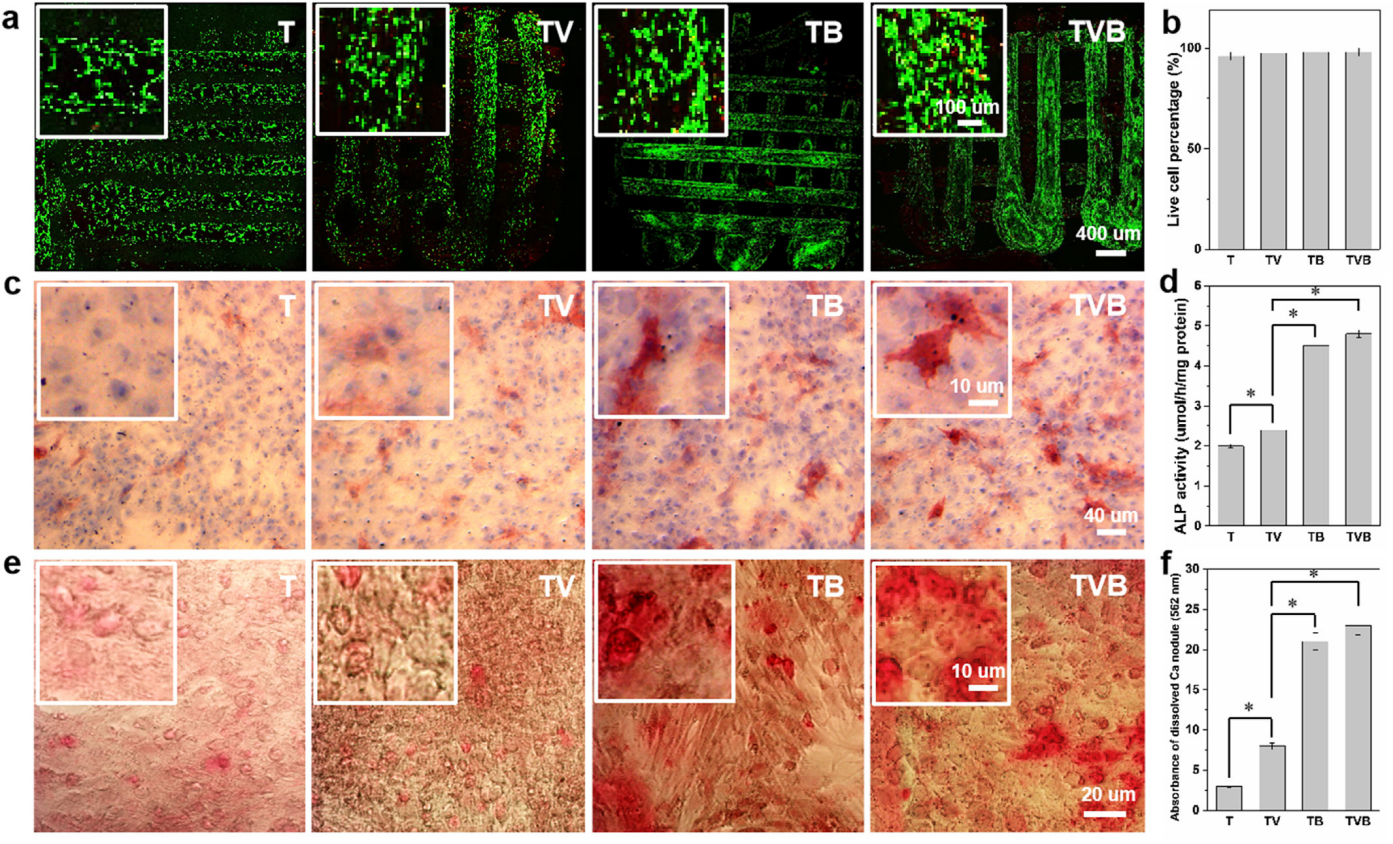
*In vitro* viability and osteogenic differentiation of rBMSCs cultured on or with scaffolds. (a–b) live and dead staining of rBMSCs on scaffolds after 3 days of culture; (c–d) ALP staining and ALP activity of rBMSCs cultured with extracts of scaffolds or on scaffolds; (e–f) Alizarin Red S staining of rBMSCs cultured with extracts of scaffolds and the quantitative analysis of the dissolved calcium nodules.

The *in vitro* osteogenic potent of scaffolds was studied by using rat bone marrow derived mesenchymal stem cells (rBMSCs) as model cells. The viability of rBMSCs cultured on different scaffolds for 3 days is shown in Fig. 5a and b. Nearly all cells were alive, showing a viability level of 98%, indicating that our scaffolds are biocompatible platforms for rBMSCs culture. As shown in Fig. 5c, after 7 days of culture with extracts of scaffolds, some ALP^+^ (color in purplish red) areas can be observed in T and TV groups, whereas significantly more ALP^+^ areas were found in TB and TVB groups. The result of ALP activity assay (Fig. 5d) also confirmed this trend, indicating that the combination of TCP presence and sustained OP release could improve the osteogenic differentiation of rBMSCs. Fig. 5e shows the calcium deposition in rBMSCs after 21 days of culture in extracts of different scaffolds. Similarly, TB and TVB groups induced significantly more formation of calcium nodules (color in red) than T and TV groups. The calcium nodules in different groups were further dissolved and their absorbance was compared (Fig. 5f). Significantly higher absorbance value was obtained for TB and TVB groups, in which TVB group induced the highest absorbance value, indicating that the dual-release of AP and OP could have a synergistic effect on rBMSC mineralization.

### 3.5 *In vivo* analyses

**Fig. 6:**
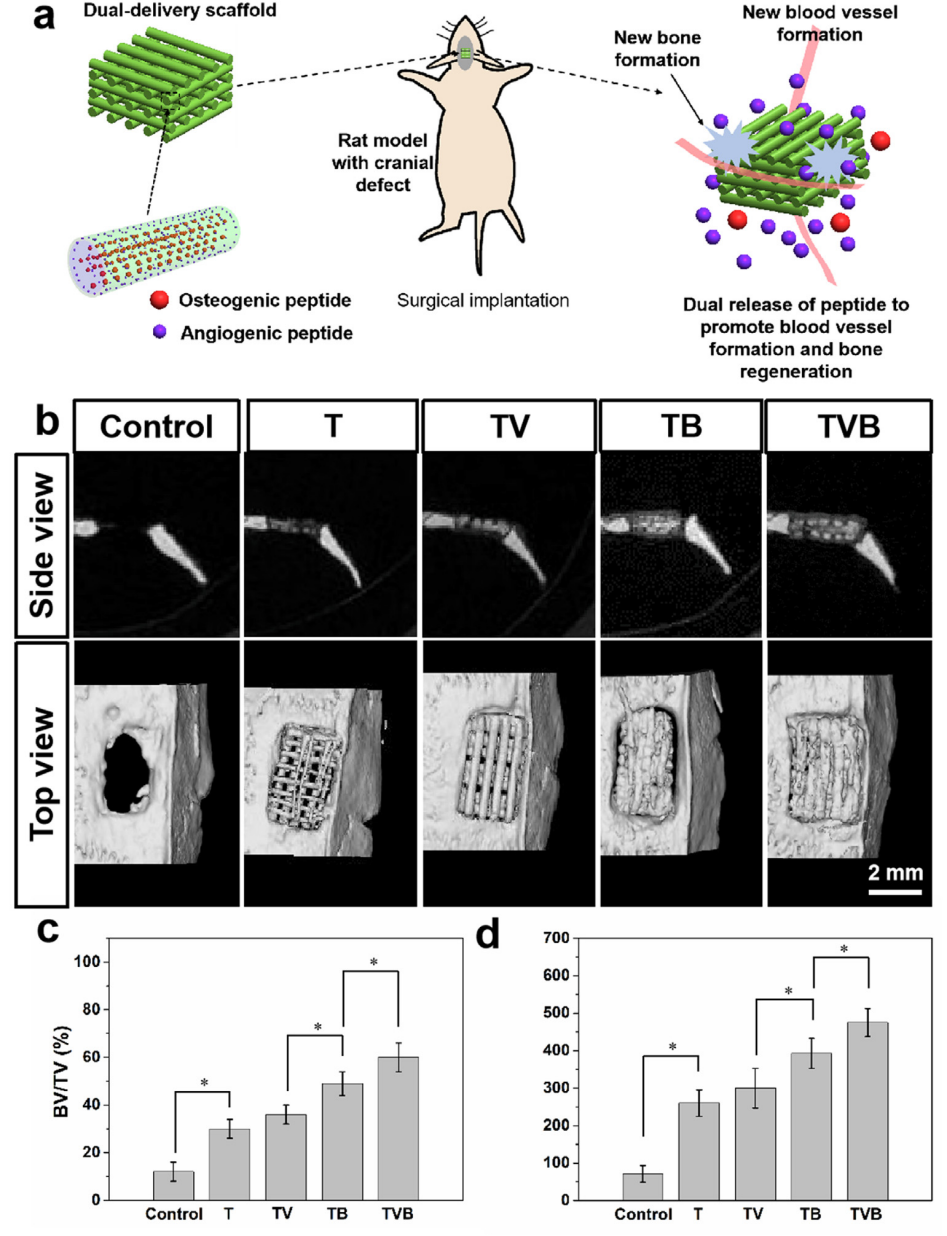
*In vivo* regeneration of rat cranial defects: (a) schematic illustration of cryogenic 3D printed dual delivery scaffolds for cranial defect mouse treatment model; (b) typical μ-CT images of regenerated cranial bone defects; (c) bone volume/total volume (BV/TV) ratio of regenerated tissue; (d) bone mineral density of regenerated bone tissue. Control group refers to non-treatment of the rat bone defect.

*In vivo* studies were also conducted to verify whether scaffolds with dual-delivery of AP and OP had any advantageous effect on bone tissue regeneration (Fig. 6a). As shown in Fig. 6b, after 3 months, bone regeneration could be only found in the peripheral part of the cranial defect of rats. For defects implanted with T and TV scaffolds, cavities among scaffold struts can still be seen, indicating an incomplete new bone formation. Differently, cavities in TB scaffolds were filled with new bone tissue but a gap still existed between the scaffold and the defect boundary. In comparison, TVB scaffolds greatly improved the bone regeneration in cranial defects, in which gaps between defect boundary and cavities in scaffolds were filled with newly regenerated bone tissue. Fig. 6c and d show the bone volume/total volume (BV/TV) ratio and bone mineral density (BMD) of different groups. It can be seen that TVB scaffolds induced the highest level of BV/TV ratio and BMD value and these could be attributed to the synergistic effect of AP and OP release-induced angiogenesis and osteogenesis.

**Fig. 7:**
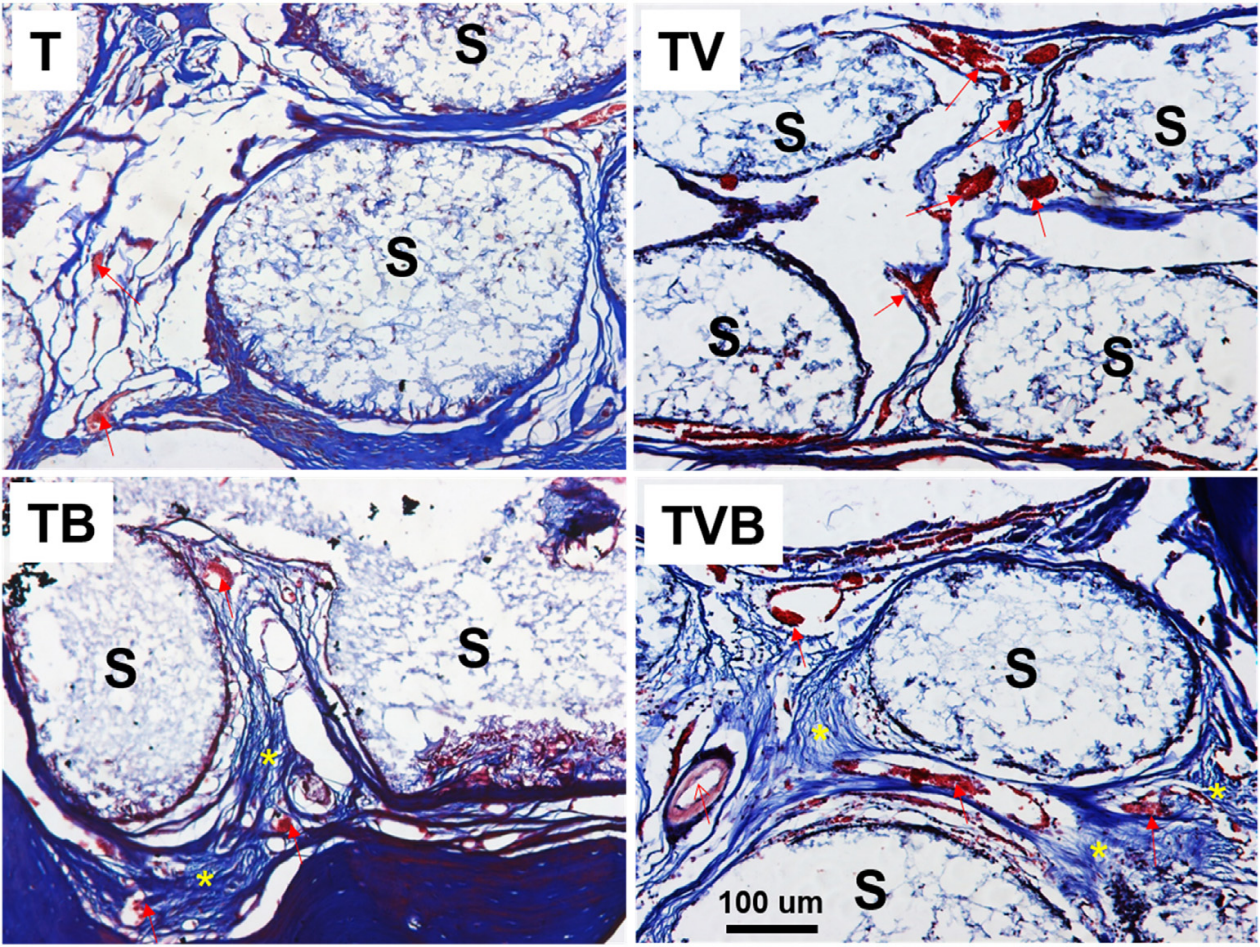
Histological analysis of regenerated tissues in the rat cranial defects 3 months after the implantation of different scaffolds through Masson's trichrome staining. Notes: “S” represents scaffold; “red arrow” indicates the vessel; “yellow star” indicates the new bone and osteoid. (For interpretation of the references to color in this figure legend, the reader is referred to the Web version of this article.)

Histological analysis of regenerated tissues in the rat cranial defects 3 months after the implantation of different scaffolds through Masson's trichrome staining (Fig. 7). After 3 months of regeneration, scaffold struts can still be observed in all groups. Limited new bone tissue and blood vessels can be observed in the holes of T scaffolds, whereas significantly more blood vessels were observed in TV group, but the new bone formation was still insufficient, indicating that the delivery of angiogenic biomolecules could greatly improve the vascularization in scaffolds during tissue regeneration, whereas angiogenic biomolecules alone cannot effectively enhance bone regeneration. In comparison, significantly more new bone formation with a better maturity was observed in TB scaffolds, in which some blood vessels were also observed, while TVB scaffolds induced not only improved new bone formation (i.e., yellow star) but also the regeneration of blood vessels with a larger diameter (i.e., red arrow), indicating that customized bone tissue engineering scaffolds with the dual-delivery of AP and OP are superior platforms to achieved both enhanced bone tissue regeneration and required vascularization.

In recent years, researchers reported several strategies to fabricate scaffolds with both osteogenic potent and angiogenic potent to realize balanced bone regeneration and vascularization. Zhang *et al.* loaded both BMP-2 and VEGF in 2-N,6-O-sulfated chitosan scaffolds [26]. By providing a sequential release of first BMP-2 and then VEGF, accelerated bone healing due to efficiently improved osteogenesis and angiogenesis was obtained. Bhattarai *et al.* Loaded BMP-2 and tauroursodeoxycholic acid (TUDCA) respectively in coaxial electrospun PLA fibers [27]. The TUDCA/BMP-2 coaxial fiber scaffolds promoted angiogenic and osteogenic differentiation in ECs and MSCs, respectively. In addition, enhanced new blood vessel formation and bone regeneration was achieved by the implantation of TUDCA/BMP-2 coaxial fiber scaffolds in rabbit calvarial defect model. Yao *et al.* developed a mesoporous silicate nanoparticles (MSNs) incorporated-3D nanofibrous gelatin (GF) scaffold for dual-delivery of BMP-2 and deferoxamine (DFO) [28]. Sustained BMP-2 release system was achieved through encapsulation into large-pored MSNs, while the relative short-term release of DFO was engineered through covalent conjugation with chitosan. The results showed that the released DFO significantly improved BMP-2-induced *in vitro* osteogenic differentiation. Zhang *et al.* developed dual-drug release bone scaffolds via alternatively printing of PCL/TCP loaded with resveratrol (RVS) and hydrogel loaded with strontium ranelate (SrRn) [29]. The 3D printed bone scaffolds containing RVS and SrRn exhibited a fast release SrRn and a relatively slow release of RVS. The results displayed that synergistic effect of RVS and SrRn contributed to osteogenic differentiation of MSCs, inhibition of osteoclast activity, and angiogenesis. In our previous investigation, multi-source multi-power electrospinning was employed to produce rhBMP-2 and rhVEGF loaded multicomponent nanofibrous scaffolds [30]. Although improved cranial bone regeneration with enhanced vascularization was achieved in mice, the electrospun multicomponent scaffolds had drawbacks such as insufficient compressive strength and limited loading level of growth factors. In comparison, our cryogenic 3D printed bone tissue engineering scaffolds with dual-delivery of AP and OP are advantageous than other bone tissue engineering scaffolds in several aspects: (1) the scaffolds are mechanically and structurally similar to human cancellous bone; (2) the addition of a large portion of TCP and the *in situ* encapsulation of OP endowed scaffolds with excellent osteoconductivity and osteoinductivity; (3) the quick AP release from the collagen I hydrogel layer improved vascularization in scaffolds via angiogenesis; (4) the presence of hydrogel layer enhanced the cell anchorage on scaffolds. To sum up, our study provides a facile method to produce customized bone tissue engineering scaffolds with improved bone forming ability and required vascularization potential, which can be readily extended to produce other complex tissue engineering scaffolds with multiple target functions.

## 4 Conclusions

In this study, customized bone tissue engineering scaffolds with dual-peptide delivery capability were successfully produced through cryogenic 3D printing of OP containing composite emulsion inks, followed by the coating of AP containing collagen I hydrogel. The dual-delivery scaffolds were mechanically similar to human cancellous bone and a sequential peptide release consisting of a quick AP release and a sustained OP release could be achieved. The dual-peptide delivery scaffolds not only improved the *in vitro* angiogenesis but also enhanced the *in vitro* osteogenic differentiation of rBMSCs. The *in vivo* results showed that the dual-peptide delivery scaffolds induced both improved new bone formation in cranial defects of rats and better vascularization in the regenerated tissues.

## Author contributions

C. Wang and Y. Tang conceived the project. C. Wang, J. Lai, K. Li and S. Zhu conducted the scaffold fabrication, characterization, *in vitro* release/degradation test and *in vitro* cell culture. K. Li and J. Liu conducted the *in vivo* experiments in rat cranial defects and analyzed the related data. C. Wang, B. Lu, J. Liu and Y. Tang, Y. Wei contributed to the writing of the manuscript.

## Declaration of competing interest

There is no conflict of interest to declare.
